# Analysis and Optimization of Machining Hardened Steel AISI 4140 with Self-Propelled Rotary Tools

**DOI:** 10.3390/ma14206106

**Published:** 2021-10-15

**Authors:** Waleed Ahmed, Hussien Hegab, Atef Mohany, Hossam Kishawy

**Affiliations:** 1 Machining Research Laboratory, Ontario Tech University, Oshawa, ON L1G 0C5, Canada; Hussien.Hegab@uoit.ca (H.H.); Hossam.kishawy@uoit.ca (H.K.); 2 Fluid-Structure Interactions Laboratory, Ontario Tech University, Oshawa, ON L1G 0C5, Canada; Atef.Mohany@uoit.ca

**Keywords:** modeling, machining, optimization, rotary tools

## Abstract

It is necessary to improve the machinability of difficult-to-cut materials such as hardened steel, nickel-based alloys, and titanium alloys as these materials offer superior properties such as chemical stability, corrosion resistance, and high strength to weight ratio, making them indispensable for many applications. Machining with self-propelled rotary tools (SPRT) is considered one of the promising techniques used to provide proper tool life even under dry conditions. In this work, an attempt has been performed to analyze, model, and optimize the machining process of AISI 4140 hardened steel using self-propelled rotary tools. Experimental analysis has been offered to (a) compare the fixed and rotary tools performance and (b) study the effect of the inclination angle on the surface quality and tool wear. Moreover, the current study implemented some artificial intelligence-based approaches (i.e., genetic programming and NSGA-II) to model and optimize the machining process of AISI 4140 hardened steel with self-propelled rotary tools. The feed rate, cutting velocity, and inclination angle were the selected design variables, while the tool wear, surface roughness, and material removal rate (MRR) were the studied outputs. The optimal surface roughness was obtained at a cutting speed of 240 m/min, an inclination angle of 20°, and a feed rate of 0.1 mm/rev. In addition, the minimum flank tool wear was observed at a cutting speed of 70 m/min, an inclination angle of 10°, and a feed rate of 0.15 mm/rev. Moreover, different weights have been assigned for the three studied outputs to offer different optimized solutions based on the designer’s interest (equal-weighted, finishing, and productivity scenarios). It should be stated that the findings of the current work offer valuable recommendations to select the optimized cutting conditions when machining hardened steel AISI 4140 within the selected ranges.

## 1. Introduction

Difficult-to-cut materials such as hardened steel, titanium alloys, nickel-based alloys, and ceramics are widely applied in many industrial fields, including aerospace, automotive, and biomedical [1]. The superior properties of these materials, as shown in Table 1, make them indispensable for many applications. However, machining of these materials is always a challenge due to their low thermal conductivity, which leads to a high concentration of the generated heat in the cutting zone and allows the temperature to hit severe levels [2]. This excessive concentrated heat affects machining performance and tool wear behavior. Additionally, instantaneous damage to the machining surface or the insert can occur due to the high temperature levels. Flood coolant is one of the widely used techniques to solve the concentrated heat problem by dissipating the generated heat to reduce the temperature. However, using the flood coolant technique has severe effects on the environment and the operator’s health [3]. The employed coolant also increases the operation cost by about 10% to 15%, as discussed by Markesberry [4]. It was found that machining using self-propelled rotary tools can be a suitable approach, especially for difficult-to-cut materials, even under dry conditions [5].

In self-propelled rotary tools, the insert is a round disc that is allowed to rotate around its axis freely. As a result of the tool motion, the whole circumference of the disc can be used as a cutting edge. The motion also allows each portion of the cutting edge to engage with the workpiece for a short time and disengage to cool down before cutting again. Therefore, a self-cooling feature occurs when the tool rotates, which dissipates the generated heat and maintains acceptable temperature levels even under dry machining conditions. Moreover, the tool wear is distributed over the whole round edge instead of being concentrated at a single point, as occurs in traditional machining. Thus, superior wear resistance was observed when machining with self-propelled rotary tools compared to traditional tools [6].

Chen et al. [9] used self-propelled rotary tools made of carbide for machining SiCw/Al composite workpieces. The results showed that rotary tools offered a dramatic increase in tool life compared to traditional tools. Ezugwu [10] observed that the use of rotary tools provided better surface roughness, reduced the machining temperature, and increased the tool life compared to the conventional cases. Wang et al. [11] have employed self-propelled rotary tools in machining Ti-6Al-4V at low cutting speeds. They showed that tool life was significantly improved, and tool wear was dramatically decreased in the case of SPRT compared to conventional cutting tools. The same observations were presented by Lei and Liu [12] when machining Ti-6Al-4V using driven rotary tools. Their results showed that tool life was increased by more than 60 times compared to the stationary round insert under the same conditions. Harun et al. [13] have also used driven rotary tools for cutting plain carbon steel. They measured the cutting-edge temperature using the thermocouple method. In addition, they modeled the thermal behavior of the cutting process using rotary tools with one-dimensional unsteady heat transfer theory. The effect of cutting and cooling conditions on tool life when machining using rotary tools was discussed by Karaguzel et al. [14]. A tool temperature model for machining using self-propelled rotary tools was developed by Kishawy et al. [15], and the results proved that lower cutting temperature occurs in the case of rotary tools compared to fixed tools. El-Mounayri et al. [16] conducted an experimental study to compare the machining characteristics (including surface quality, tool wear, and cutting forces) when utilizing self-propelled rotary with conventional tools. The hardness of the workpiece material was 55RC workpiece, and coated carbide inserts were used. The results showed that SPRT provided better overall performance compared to traditional tools. Moreover, Kishawy et al. [17] developed an analytical model to predict the chip flow angle for the tube-end turning machining process of hardened steel using self-propelled rotary tools. This work was further developed, and another analytical model was provided to accurately predict the cutting forces when machining with self-propelled rotary tools [18]. Another analytical model was developed by Kishawy et al. [19] to accurately predict the cutting forces and tool rotational speed for machining with self-propelled rotary tools by considering the bearing friction. The performance of machining hardened steel with self-propelled rotary tools was analyzed by Kishawy and Wilcox in terms of tool wear and chip morphology [20]. Recently, Thellaputta et al. [21] studied the effect of different machining variables on the milling process performance of Inconel 625 using rotary tools. In this study, an infrared thermal camera was utilized to measure the cutting temperature. It was observed that the machining temperature increased as the cutting speed and feed rate increased. The effect of different cutting conditions on the cutting forces and surface roughness when machining hardened 41Cr4 steel with SPRT was investigated by Nieslony et al. [22]. Furthermore, Ahmed et al. [23,24] performed numerical and experimental studies to model and investigate the machining process with self-propelled rotary tools, and 3-D distribution of the tool’s temperature was presented.

On the other hand, multi-objective optimization using the non-sorted genetic algorithm (NSGA-II) was employed in the study by Abbas et al. [25] to optimize the machining performance and sustainability aspects using different cooling techniques when machining AISI 1045 steel. An integrated approach between the genetic algorithm and neural network was used by Sangwan et al. [26] to optimize the machining variables to minimize the surface roughness when machining Ti-6al-4v. Pawar et al. [27] employed the artificial bee colony algorithm to perform multi-objective optimization for the wire-electric discharge process. The tool wear, surface roughness, and productivity aspects were considered as machining outputs. Dabade et al. [28] utilized self-propelled rotary tools for the face milling process. Optimization was employed to study the effect of the machining variables on the output responses, including chip cross-surface area and surface roughness. The inclination angle was the most significant variable that affected the machining outputs. Hao et al. [29] used the artificial neural network (ANN) technique to predict the cutting force components when machining low carbon steel. Cutting velocity, feed rate, tool inclination angle, and depth of cut were considered as input variables. Nguyen et al. [30] developed a sustainability-based optimization model for the turning process of hardened steel using rotary tools. The genetic algorithm was used, aiming to reduce the machining cost and surface roughness, as well as enhance energy efficiency and operation safety.

**Figure 1:**
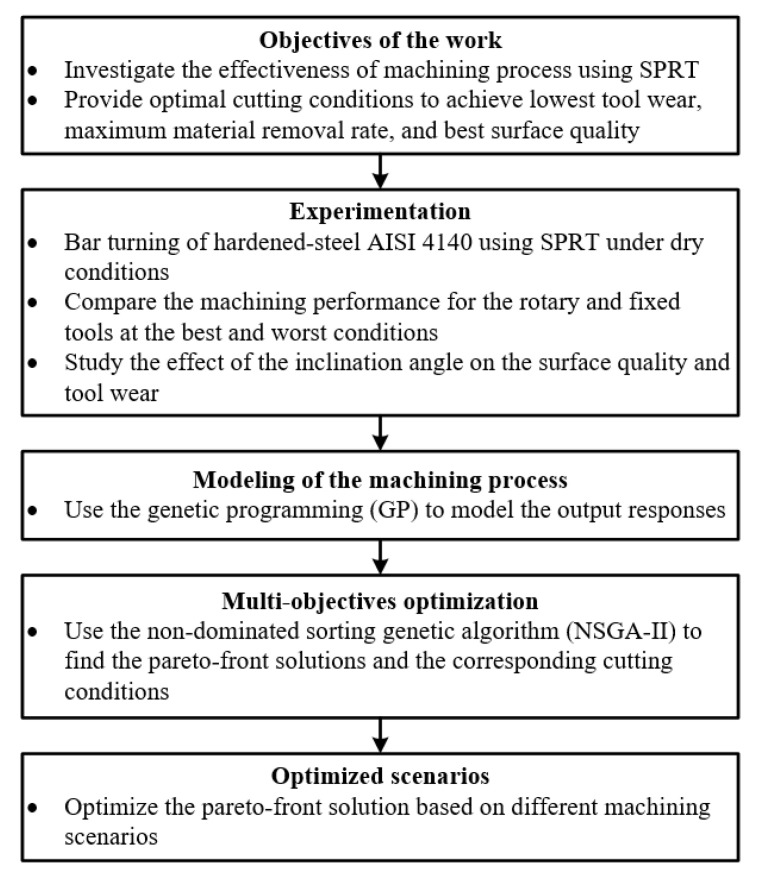
Flow chart for the research methodology.

There are minimal studies in the open literature that implemented the multi-objective optimization approach in the area of machining with self-propelled rotary tools [31,32]. In addition, few models have been performed (either analytical or artificial intelligence-based models) to model wear behavior and surface integrity when machining with rotary tools; the majority of the developed models are focused on the cutting forces. Firstly, in this work, experimental analysis has been offered to (a) compare between the fixed and rotary tools performance and (b) study the effect of the inclination angle on the surface quality and tool wear. Secondly, the current study implemented some artificial intelligence-based approaches (i.e., genetic programming and NSGA-II) to model and optimize the machining process of hardened steel AISI 4140 with self-propelled rotary tools. The feed rate, cutting velocity, and inclination angle were selected to be the design variables, while the tool wear, surface roughness, and material removal rate (MRR) were the studied outputs. Moreover, different weights have been assigned for the three studied outputs to offer different optimized solutions based on the designer’s interest (equal-weighted, finishing, and productivity scenarios). Figure 1 shows the flow chart of the current work methodology.

## 2. Materials and Methods

In the current study, different cutting tests were carried out to investigate and analyze the effect of the cutting conditions on machining performance when using self-propelled rotary tools. The workpiece material was hardened steel AISI 4140 (46 ± 2 HRC). The hardened steel AISI 4140 is widely used in many industrial applications, including shafts, driving pins, axles, link components, gears, and milling spindles. That is attributed to its high resistance to wear, corrosion, and abrasion, as well as its high durability, compared to untreated steel. However, machining hardened steel is a challenge due to the relatively high hardness, which causes abrasive wear and accordingly shortens tool life, especially under dry conditions.

**Figure 2:**
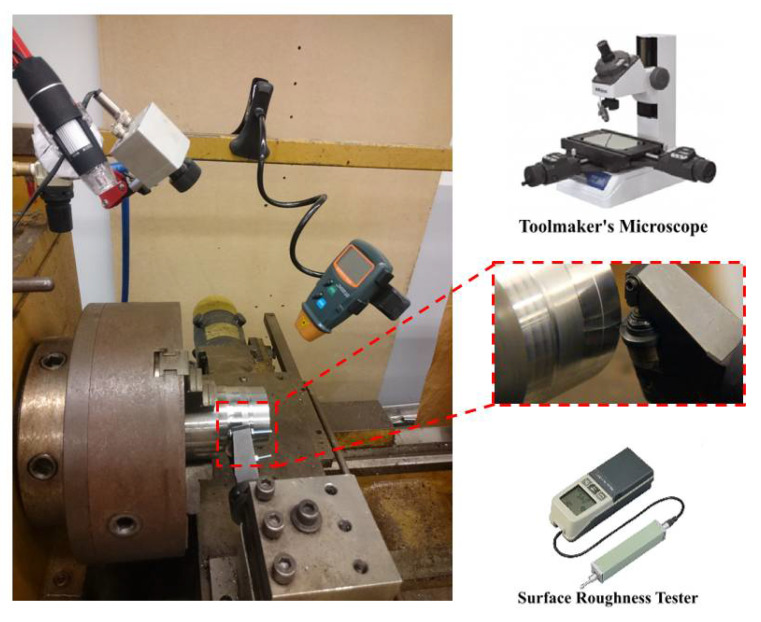
Experimental setup for the turning process using a self-propelled rotary tool.

A tube-shaped workpiece was used to achieve homogeneous properties during the heat treatment process. The outer diameter of the workpiece was 100 mm, while the inner diameter was 50 mm. Table 2 shows the chemical composition of AISI 4140 steel. A carbide round insert with an outer diameter of 27 mm was also used. The rake angle was −5°, while the clearance angle was 5°. Figure 2 shows the experimental setup of the current study.

**Figure 3:**
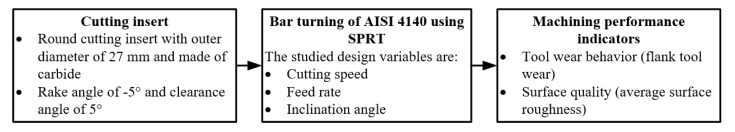
Flow chart of the experimental procedures.

The feed rate, cutting speed, and inclination angle were selected as design variables, while the average surface roughness and flank tool wear were chosen to be performance indicators. The average surface roughness was used to evaluate the machined surface quality. The arithmetical mean deviation of the assessed profile (*Ra*) was measured using Mitutoyo (SJ.201) portable surface roughness at a cut-off length of 2.5 mm. The surface roughness was measured at three different locations, and the average value was calculated and used for the analysis. Mitutoya toolmaker’s microscope (TM-A505B) was used to measure the average flank wear of the insert after each run. The flank tool wear was measured at four different locations on the circular flank face of the insert, and the average value was obtained and used in the analysis. Figure 3 shows a flow chart for the experimental procedures.

Taguchi’s approach was utilized in the current study to conduct a minimum number of experiments. Three design variables with four levels each were used in the present study. The selected design variables were inclination angle (*i*), feed rate (*f*), and cutting speed (*V*). Three design variables with four levels each (i.e., 4^3^) were utilized; therefore, the full L64OA orthogonal array should be used. However, a fractional factorial L16OA orthogonal array was employed to save cost and time [33]. The levels for the three design variables were selected to be (a) 5, 10, 15, and 20° for the inclination angle; (b) 0.1, 0.15, 0.2, and 0.25 mm/rev for the feed rate; (c) 70, 127, 167, and 240 m/min for the cutting speed. The levels of each design variable were selected based on the recommendation of the tool’s manufacturer as well as the machine tool capabilities. The depth of cut was 0.2 mm, while the cutting length was 100 mm for all runs. Table 3 shows the 16 experiments of the current study.

## 3. Results and Discussion

Table 4 shows the results of the average flank tool wear and average surface roughness during dry machining with self-propelled rotary tools. The minimum flank tool wear was observed at test 6, where the cutting speed was 70 m/min, the inclination angle was 10°, and the feed rate was 0.15 mm/rev. Test 14 showed the highest flank tool wear, where the cutting speed was 167 m/min, the inclination angle was 20°, and the feed rate was 0.15 mm/rev. In general, the results showed that reducing the cutting speed leads to low flank wear, as expected. When using rotary tools, it was found that increasing the feed rate decreases flank wear. That could be attributed to the fact that the cutting process becomes more stable at a high feed rate as continuous chip was observed; however, discontinuous chip was noticed at a low feed rate.

Regarding the average surface roughness, the results revealed that the variation of the cutting conditions had a corresponding effect on the surface roughness. The optimal surface roughness was obtained at test 13, where the cutting speed was 240 m/min, the inclination angle was 20°, and the feed rate was 0.1 mm/rev. It was observed that increasing the cutting velocity led to a reduction in the surface roughness value, as expected. Besides, increasing the feed rate led to a deterioration of the machined surface due to the increase of the chip load.

**Figure 4:**
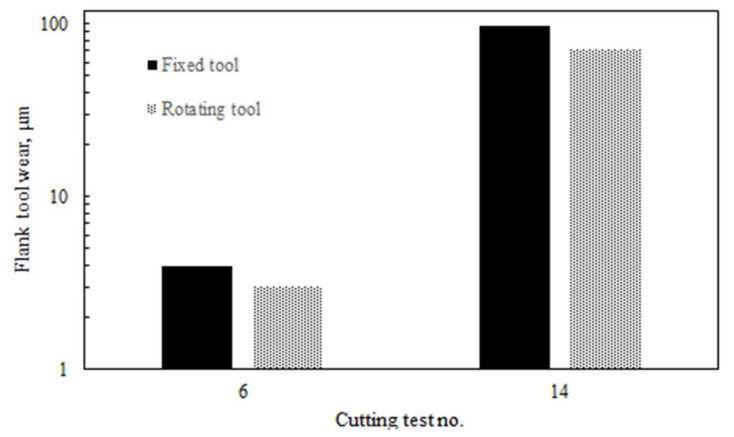
Tool wear results at worst and best scenarios for rotary tool versus fixed tool.

A comparison between the fixed and rotary tools was performed to study the effect of the tool motion on the studied machining responses. Figure 4 shows the tool wear results for fixed and rotary tools at the best and worst conditions (i.e., test 6 and test 14). The wear of the rotary tool was reduced by 37% at test 14 (where the maximum tool wear occurred) compared to the fixed tool. At test 6, the tool wear of the rotary tool was reduced by 22% compared to the fixed tool. That could be attributed to the benefits of the tool rotational motion.

**Figure 5:**
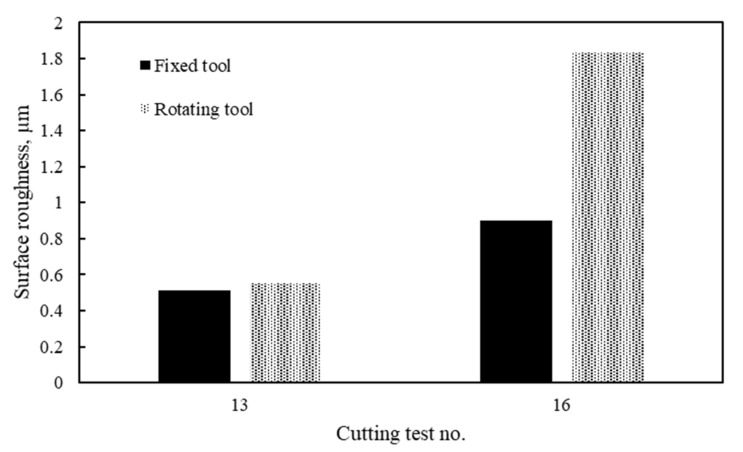
Average surface roughness results at worst and best scenarios for rotary tool versus fixed tool.

Figure 5 shows surface roughness results for fixed and rotary tools at the best and worst conditions (i.e., test 13 and test 16). In general, the surface roughness values of rotary tools are relatively low compared to conventional tools (i.e., single point) due to the large radius of the round insert compared to the nose radius of the conventional tool. However, better surface roughness was provided by fixed round tools compared to round tools under motion, especially at the worst condition (i.e., test 16), where the surface roughness of the rotary insert achieved 1.83 µm. That could be due to different possible factors, including machining stability, which is significantly affected by the dynamic nature of the rotary tool. This is because the self-propelled rotary tool is allowed to freely rotate around its axis due to the chip tangential force which guides the tool motion. One of the solutions to improve the surface roughness when using self-propelled rotary tools is to enhance the design of the tool holder by increasing its rigidity. The surface roughness is also affected by the generated marks in the direction of the relative cutting velocity as a result of the tool motion, as discussed in the previous work [34].

**Figure 6:**
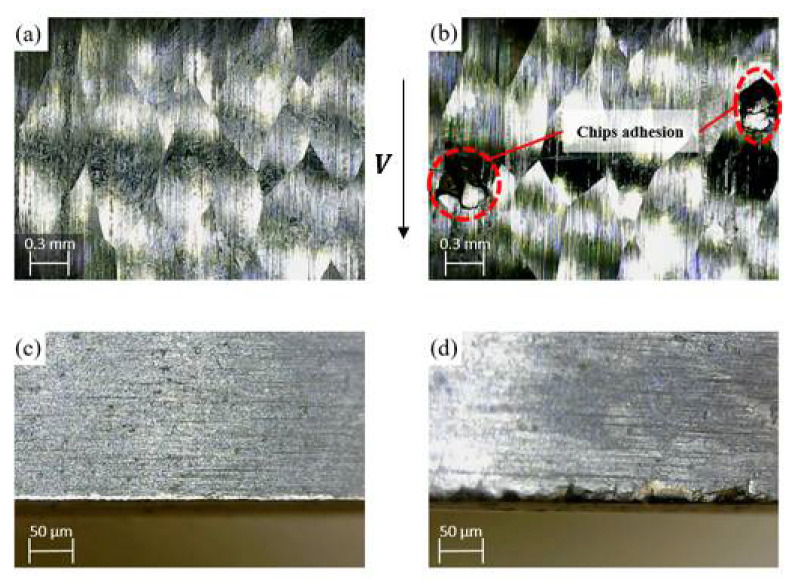
A Comparison between the finished surface and corresponding tool damage based on the inclination angle; (**a**), and (**c**) at 20° and (**b**), and (**d**) at 5°.

To study the effect of the inclination angle on the tool wear and the surface quality, a comparison was performed between two cutting tests under certain conditions of cutting speed and feed rate (i.e., *V* = 167 m/min and *f* = 0.15 mm/rev) with two different inclination angles of 5° and 20°, as shown in Figure 6. It was observed that when using a low inclination angle (i.e., 5°), the chips collided and were pushed into the workpiece surface, as shown in Figure 6b. Afterward, the cutting edge crushes the adhered chips, which increases tool wear, as can be seen in Figure 6d. On the other hand, no chip adhesion was observed in the machined surface at a 20° inclination angle (see Figure 6a), and accordingly, lower tool wear was obtained compared to the case of 5° inclination angle (see Figure 6c). That can be attributed to the increase in the chip flow angle based on the oblique cutting principles, as confirmed by Yamamoto et al. [35].

## 4. Modeling of the Machining Characteristics

In this section, genetic programming (GP) was used to empirically model surface roughness (Ra) and tool wear (VB). Genetic programming is considered one of the most effective artificial intelligence techniques, and it is used in different engineering applications [36]. In genetic programming, each program is built of a tree structure of terminals and functions (i.e., genotype). The terminals (i.e., leaves) are the inputs to the program, and the used functions of the GP program include mathematical functions, programming functions, and arithmetic operations. Every generated model is presented as a chromosome, and the fitness function is used to evaluate each chromosome. The fitness function measures the error between the model output and the input data. Genetic operators include mutation, and crossover factors are then utilized to generate new chromosomes. In the current study, the Eureqa software was used to develop models of the surface roughness and tool wear, as shown in Equations (1) and (2), respectively. It should be stated that the currently developed models are based on non-linear regression, and they are valid within the selected ranges for the studied design parameters. In addition, the current technique (genetic programming) was used in different studies to model the machining performance [37,38,39,40].

(1) $$Ra=\;0.0066*i^{2}+42.91*i*f^{4}-0.07*i-2.20e^{-9}*V*i^{5}-10.9*i*f^{3}+1.12\;$$

(2) $$\;\begin{array}{cc} VB & =387.22+\frac{8.32}{f}+16.53*i*f+0.03*f*V^{2}\\ & -\frac{21799.6+16.53*i^{2}*f^{2}}{V}-2.31*V-50.67*V*f^{3} \end{array}$$

**Figure 7:**
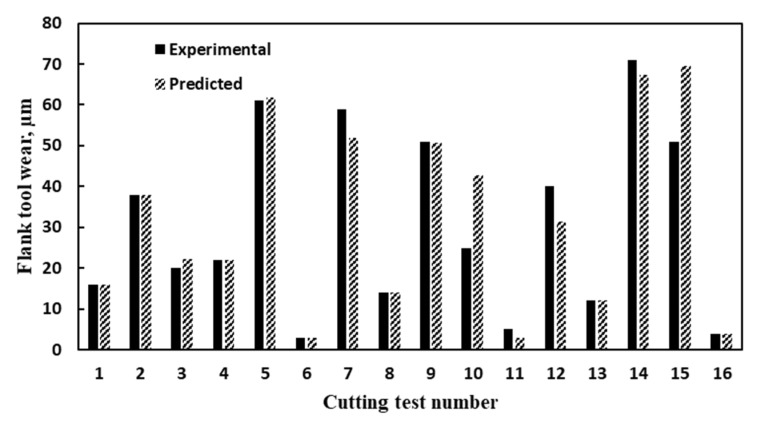
A Comparison between experimental and predicted flank tool wear.

**Figure 8:**
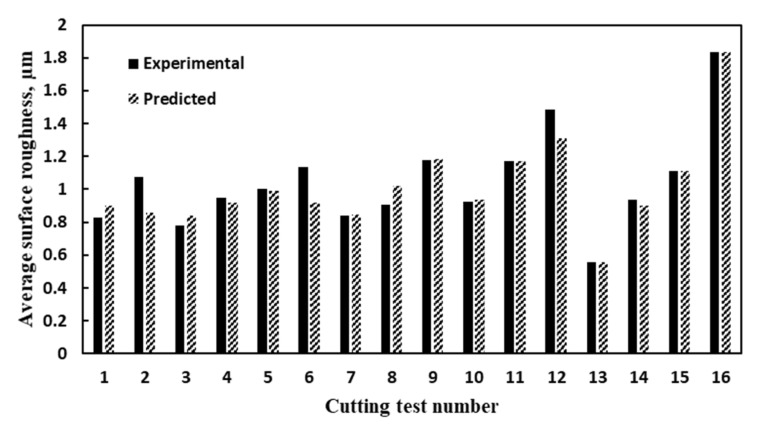
A Comparison between experimental and predicted average surface roughness.

Figure 7 and Figure 8 show a comparison between the experimental and predicted results for flank tool wear and average surface roughness, respectively. The surface roughness (*Ra*) model showed average model accuracy of 94.33% with 0.89 goodness of fit (*R*^2^) and 0.06 mean absolute error. Besides, the average model accuracy of 87.44% was achieved for the tool wear model with 0.88 goodness of fit (*R*^2^) and 3.82 mean absolute error.

## 5. Multi-Objective Optimization

The non-dominated sorting genetic algorithm (NSGA-II) was used in the current study to perform multi-objective optimization of the generated models. The NSGA-II is one of the popular multi-objective optimization techniques as it utilizes special features such as fast non-dominated method, fast crowded estimation of distances approach, and simple operator to perform a crowded comparison to find the optimal Pareto-front solutions [41]. The genetic algorithm evolutionary operators such as crossover and mutation are utilized in the NSGA-II algorithm. The general steps of the NSGA-II can be summarized as follows [42]:

- Select the size of the population based on the constraints and their range;
- Perform non-dominated sort for the initialized populations;
- Assign crowding distance values for the population of individuals;
- Select the individuals based on the rank and the crowding distance;
- Apply the genetic algorithm crossover and mutation operators;
- Recombine and select an individual for the next generation until the population size exceeds the current size.

Three machining objective functions were considered in the optimization process; tool wear, surface roughness, and material removal rate. It should be stated that the generated models obtained in Section 3 were used as objective functions in this stage. The problem constraints, according to the current experimental plan, are as follows:

$$\left\{\begin{array}{c} 5{}^{\circ}\leq i\leq 20{}^{\circ}\\ 0.1\;\mathrm{mm}/\mathrm{rev}\leq f\leq 0.25\;\mathrm{mm}/\mathrm{rev}\\ 70\;m/\min\leq V\leq 240\;m/\min \end{array}\right.$$

**Figure 9:**
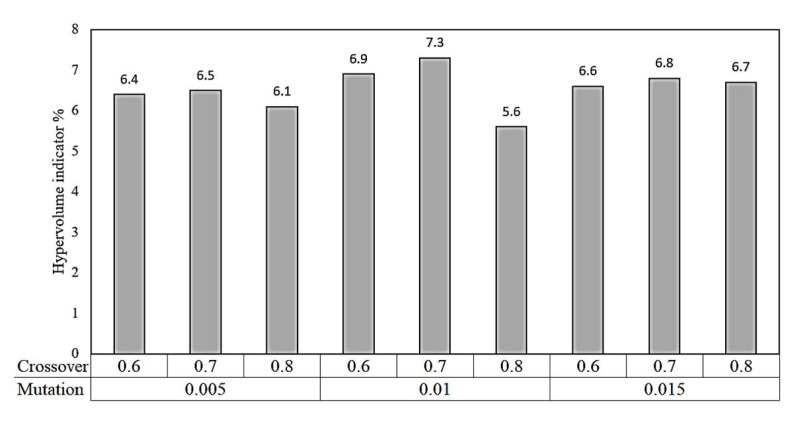
Hypervolume indicator for various values of mutation and crossover parameters.

A sensitivity analysis was performed to select the optimized parameters used for NSGA-II. Hypervolume Indicator was calculated to evaluate the performance of the Pareto-front solutions set. This indicator is used to measure the convergence and the diversity of Pareto-front solutions [43]. The hypervolume indicator follows the higher-better criteria, which means the optimized solution can be found at the highest hypervolume indicators. In the current study, the hypervolume indicator was calculated at three different values of crossover (i.e., 0.6, 0.7, and 0.8) and three values of mutation (i.e., 0.005, 0.01, and 0.015). Figure 9 shows that the highest hypervolume indicator of 7.3% was obtained at a mutation factor of 0.01 and a crossover rate of 0.7, which was used in the current optimization algorithm. The population size of 400 was selected, and the solver was allowed to proceed until the function tolerance of 10^−4^ was achieved.

**Figure 10:**
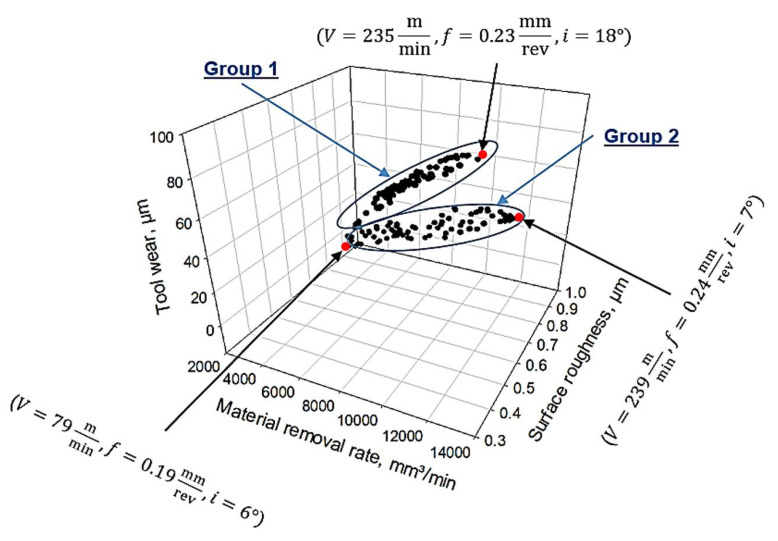
Pareto-front solutions.

Figure 10 shows the Pareto-front solutions for the three objectives functions. The Pareto-front solution is distributed into two groups, group 1 and group 2. For the first group, it can be noticed that the dominant relationship between the tool wear and the material removal rate is an interdependent based-relation. That could be attributed to the high cutting velocity, which associates with the high material removal rate. Besides, there is a reduction in the surface roughness values when decreasing the inclination angle and the cutting speed. That is because the surface roughness when machining with self-propelled rotary tools depends on the machining stability (the dynamic nature of the process). The tool rotational speed is directly affected by the cutting velocity and the inclination angle, as seen in Equation (3) [44]:

(3) $$V_{r}=V\sin\left(i\right)$$

where $V_{r}$ is the tool rotational speed, V is the cutting velocity, and i is the inclination angle.

Therefore, the low levels for both inclination angle and cutting velocity lead to the slow rotational speed of the cutting insert, which increases the machining stability and produces better surface quality. For the second group, the dominant relationship is an interdependent relationship between the surface roughness and the material removal rate. The higher values of the surface roughness at the high material removal rate are due to the large value of the cutting velocity, which increases the rotational speed of the insert (see Equation (3)) and accordingly reduces the machining stability. It should be stated that the three selected points in Figure 10 represent the optimal boundaries of the obtained two groups.

## 6. Optimized Scenarios

This section provides a new approach to optimize Pareto-front solutions based on different machining scenarios. Three different machining scenarios were investigated, namely: equal-weighted, productivity, and finishing. Different weighting factors were assigned to each scenario to evaluate the output responses. It should be stated that the weights provided in each studied scenario were based on a specific design requirement; for example, 60% is assigned to MRR in the case of the productivity scenario, while 70% is assigned to Ra in the case of the finishing scenario. In this way, this optimized approach can allow different weight coefficients based on the desire requirements of the decision-maker. Table 5 represents the scenarios used and the weighting factors for each scenario. The multi-objective optimization NSGA-II provides some Pareto-front solutions, and to choose the best optimal solution for a certain scenario (e.g., finishing or productivity), normalized Pareto-front solutions based on each scenario were obtained. Afterward, the highest normalized solution in each scenario was considered. The highest optimized solution means that the selected solution achieves the best balance between all the output responses within each studied scenario.

Table 6 shows the result of the normalized optimal solutions for the three studied scenarios. The optimal cutting conditions for the productivity scenario were obtained at the highest cutting velocity and feed rate (i.e., *V* = 240 m/min and *f* = 0.25 mm/rev), as expected, and at an inclination angle of 7°. In contrast, the optimum conditions for the finishing scenario were found at a cutting velocity of 235 m/min, a feed rate of 0.19 mm/rev, and an inclination angle of 19°. Moreover, the optimal conditions for the equal-weighted scenario) were found at a cutting velocity of 98 m/min, a feed rate of 0.23 mm/rev, and an inclination angle of 7°.

**Figure 11:**
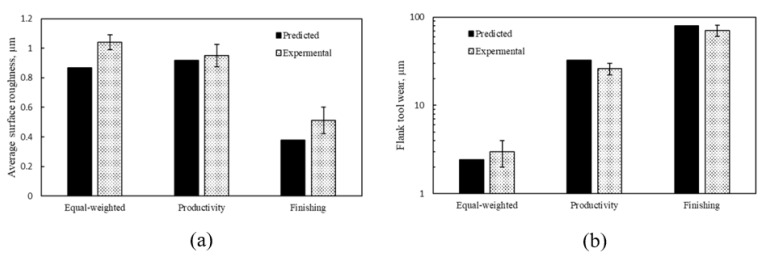
Experimental validation of the results of the optimized scenario; (**a**) Average surface roughness and (**b**) Flank tool wear.

To validate the effectiveness of the three studied scenarios, confirmation experimental tests were conducted, and the results showed good agreement with the predicted values, as can be seen in Figure 11. For the flank wear results, the maximum deviation was about 8 µm at the finishing scenario. Regarding the surface roughness results, the maximum deviation was about 0.17 µm for the equal-weighted case.

## 7. Conclusions and Future Work

This work offers an attempt to analyze, model, and optimize the machining of AISI 4140 hardened steel with SPRT. The main findings obtained in this study have been summarized as follows:

- Using a self-propelled rotary tool reduced the flank tool wear by 37% and 22% at the worst and best cutting conditions, respectively, compared to the fixed tool;
- Unlike conventional cutting, increasing the feed rate led to a decrease in the flank tool wear;
- A comparison between the self-propelled rotary tool and the fixed tool shows that the fixed tool provided better surface roughness;
- A comparison between two cutting tests with different inclination angles shows that there were no chips adhesion observed in the machined surface at 20° inclination angle, and accordingly, lower tool wear was obtained compared to the case of 5° inclination angle;
- The surface roughness values of rotary tools are relatively low compared to conventional tools (i.e., single point) due to the large radius of the round insert compared to the nose radius of the conventional tool. However, better surface roughness was provided by fixed round tools compared to the round tools under rotational motion;
- Based on the optimized scenarios of multi-objective optimization (NSGA-II), the optimal cutting variable levels for the equal-weighted scenario were found at a cutting velocity of 98 m/min, a feed rate of 0.23 mm/rev, and an inclination angle of 7°. Besides, the optimal cutting conditions for the productivity scenario were obtained at the highest cutting velocity and feed rate (i.e., *V* = 240 m/min and *f* = 0.25 mm/rev), and an inclination angle of 7°. While the optimum conditions for the finishing scenario were found at a cutting velocity of 235 m/min, a feed rate of 0.19 mm/rev, and an inclination angle of 19°;
- To validate the effectiveness of the three studied scenarios, confirmation experimental tests have been conducted, and the results showed a good agreement with the predicted values.

It should be stated that the findings of the current work offer valuable recommendations to select the optimized cutting conditions when machining hardened steel AISI 4140. In terms of future work, an in-depth analytical model is needed to fully understand the chip formation mechanisms for the machining process with self-propelled rotary tools. In addition, the effect of the machining parameters (cutting speed, inclination angle, feed rate) on the microstructure of the generated machined surface when using rotary and conventional tools should be studied. Furthermore, to investigate the durability aspect, a progressive tool wear test followed by tool wear mechanism analysis should be conducted for both rotary and conventional tools.

## Figures and Tables

**Table 1:** Properties of some super-alloys at room temperature [7,8].

Property	Material			
	Ti-6Al-4 V	Inconel 718	Titanium	AISI 4140
Density (g/cm^3^)	4.43	8.22	4.5	7.85
Ultimate tensile strength (MPa)	950	1350	220	729.5
Yield strength (MPa)	880	1170	140	379.2
Modulus of elasticity (GPa)	113.8	200	116	198
Ductility (%)	14	16	54	25.7
Fracture toughness (MPa m^1/2^)	75	96.4	70	66
Thermal conductivity (W/mK)	6.7	11.4	17	42.7

**Table 2:** Chemical composite (wt. %) of AISI 4140.

C	SI	Mn	Cr	Mo	Fe
0.38%–0.43%	0.15%–0.3%	0.7%–1%	0.8%–1.1%	0.15%–0.25%	96.75%–97.84%

**Table 3:** The design of experiments for the machining runs.

Test No	Inclination Angle Levels	Feed Rate Levels	Cutting Speed Levels
1	1	1	1
2	1	2	2
3	1	3	3
4	1	4	4
5	2	1	2
6	2	2	1
7	2	3	4
8	2	4	3
9	3	1	3
10	3	2	4
11	3	3	1
12	3	4	2
13	4	1	4
14	4	2	3
15	4	3	2
16	4	4	1

**Table 4:** Average surface roughness (*Ra*) and tool wear (*VB*) results, where *(i*) is the inclination angle, (*f*) is the feed rate, and (*V*) is the cutting speed.

Test No	*i* (°)	*f* (mm/rev)	*V* (m/min)	*VB* (µm)	*Ra* (µm)
1	5	0.1	70	16	0.83
2	5	0.15	127	38	1.08
3	5	0.2	167	20	0.78
4	5	0.25	240	22	0.95
5	10	0.1	127	61	1.00
6	10	0.15	70	3	1.13
7	10	0.2	240	59	0.84
8	10	0.25	167	14	0.90
9	15	0.1	167	51	1.18
10	15	0.15	240	25	0.93
11	15	0.2	70	5	1.17
12	15	0.25	127	40	1.48
13	20	0.1	240	12	0.56
14	20	0.15	167	71	0.94
15	20	0.2	127	51	1.11
16	20	0.25	70	4	1.83

**Table 5:** The weighting factors for the machining scenarios.

Scenario	Machining Outputs		
	*(Ra)*	*(VB)*	*(MRR)*
(A): Equal-weighted	33.33%	33.33%	33.33%
(B): Productivity	10%	30%	60%
(C): Finishing	70%	20%	10%

**Table 6:** A summary of the optimal solutions for the studied scenarios.

Scenario	Machining Outputs		
	*Ra* (µm)	*VB* (µm)	*MRR* (mm^3^/min)
(A): Equal-weighted	0.87	2.42	4580
(B): Productivity	0.92	32.56	11,851
(C): Finishing	0.38	79.93	9156

## Data Availability

Not applicable.
